# Harnessing the Power of AI to Improve Detection, Monitoring, and Public Health Interventions for Japanese Encephalitis

**DOI:** 10.3390/biomedicines13010042

**Published:** 2024-12-27

**Authors:** Junhua Xiao, Evie Kendal, Faith A. A. Kwa

**Affiliations:** School of Health Sciences, Swinburne University of Technology, Hawthorn, VIC 3122, Australia; jxiao@swin.edu.au (J.X.); fkwa@swin.edu.au (F.A.A.K.)

**Keywords:** Japanese Encephalitis Virus, JEV, Japanese Encephalitis, JE, artificial intelligence, AI

## Abstract

Japanese Encephalitis (JE) is the leading cause of viral encephalitis in regions with endemic Japanese Encephalitis Virus (JEV) infections. Background/Objectives: The aim of this review is to consider the potential role of artificial intelligence (AI) to improve detection, monitoring and public health interventions for JE. Discussion: As climate change continues to impact mosquito population growth patterns, more regions will be affected by mosquito-borne diseases, including JE. Improving diagnosis and surveillance, while continuing preventive measures, such as widespread vaccination campaigns in endemic regions, will be essential to reduce morbidity and mortality associated with JEV. Conclusions: With careful integration, AI mathematical and mechanistic models could be useful tools for combating the growing threat of JEV infections globally.

## 1. Introduction

Japanese Encephalitis (JE) is a mosquito-borne tropical disease caused by infection with Japanese Encephalitis Virus (JEV). Endemic throughout most of Asia and various locations within the Western Pacific region, the disease has been spreading southward over recent years due to the effects of climate change [1], including a recent outbreak in Southeastern Australia in 2022 [2,3]. The emergence of JEV and other neurotropic flaviviruses in immunologically naïve populations has led to updated diagnostic protocols and calls for better surveillance mechanisms [4,5]. This review considers the potential role of artificial intelligence (AI) in this endeavor, addressing both practical and ethical issues.

The current study is significant, as better public health management of JEV has the potential to greatly reduce morbidity and mortality associated with this infection. JE is the leading cause of viral encephalitis in endemic regions, with an estimated 70–100,000 clinical cases and 15–25,000 fatalities globally per annum [6,7]. Most individuals infected with JEV will have mild symptoms that resolve over time, including low-grade fever, nausea, diarrhea, headaches, and muscle pains, with some cases remaining asymptomatic. However, in a minority of infections, the virus can enter the central nervous system, leading to more severe symptoms, such as seizures and altered consciousness or behavior, and can lead to long-term neurological and psychiatric damage [8]. As such, improving timely diagnosis and surveillance, while continuing current prevention strategies, including vaccination programs in affected regions, is essential. In this review, we aim to identify some of the promises and perils of engaging machinic learning for JEV management, including in regions that have not previously seen locally acquired infections.

## 2. Importance and Challenges with Current Surveillance of JEV

There are various ways to diagnose a JEV infection. Virus detection in cerebrospinal fluid (CSF) and serum of suspected patients using real-time polymerase chain reaction (RT-PCR) is one method, but it may not always be an efficient diagnostic test due to the virus’s transient presence and the low levels of viral genetic material in these bodily fluids that usually become undetectable soon after the onset of symptoms [9]. Now, in most countries, the surveillance of JE is routinely performed using enzyme-linked immunosorbent assay (ELISA), which detects the presence of anti-JEV immunoglobulin M in the CSF or serum of individuals with a suspected infection [10]. However, the quality control measures and performance of commercially available ELISAs vary in their sensitivity and specificity [9]. This questions the reliability of the reported incidence of JE and its trends, as not all true cases may be captured.

To address these concerns, molecular methods, including droplet digital PCR (ddPCR), have been further developed and refined to improve the sensitivity and accuracy of JEV detection [11]. This approach has a superior sensitivity, as the absolute number of target viral RNAs in a range of biological samples, such as visceral tissues, CSF, semen, and blood, can be established from the ratio of positive events to total partitions using binomial Poisson statistics [11]. Wu et al. (2017) reported that the detection limit for ddPCR was almost 100-fold greater in sensitivity than earlier conventional RT-PCR methods, leading to a 10% increase in positive cases detected by the former [11]. In more recent times, quantitative duplex RT-PCR (dRRT-PCR) was developed to simultaneously detect and differentiate flaviviruses from the JE serocomplex [12]. This advanced molecular approach requires two primers specific to identifying specific JEV subtypes and other classes of flaviviruses. Once the unknown viral genome sequence is established, referencing this sequence against known full-length genome sequences of the JE serocomplex viruses published in GenBank is required, thus allowing for rapid detection of explicit JEV subtypes causing infection outbreaks in humans and animal hosts [12]. The complex nature of the epidemiology of JEV and the existence of variants across different geographical locations continue to challenge the accuracy and effectiveness of current surveillance strategies. 

Surveillance of JE is essential in ascertaining its epidemiology, defining populations at risk, predicting the burden of outbreaks in a country, and establishing the geographical spread and scale of transmission [13]. This information is crucial for policy makers to make informed decisions surrounding JE vaccination programs, including prioritizing vulnerable and age-specific groups, and in the allocation of public health resources. Common surveillance measures that can overcome the limitations of JEV detection during a short viremic period include serosurveillance, where paired acute and convalescent sera of suspected infected individuals can be used to delimit geographical locations at risk [14]. Such testing should not only be conducted in humans but also in the mosquito populations, animal hosts, and other species (e.g., wild birds) in affected areas [14]. For serosurveillance techniques to fulfil the objectives of JE surveillance, care must be taken in analyzing the intimate relationship between the antigens of flaviviruses and establishing their cross-reactivity [15]. In addition, the identity of the specific JEV variant in any index case should be validated using the plaque-reduction neutralization test (PRNT) that can help to discriminate between group reactive antibodies [16]. The level of sensitivity and specificity of the various diagnostic tests will need to be scrutinized when selecting one or more to accomplish reliable targeted and risk-based surveillance of JEV infections and outbreaks [14]. The World Health Organization has stipulated that JE surveillance must be conducted on all age groups throughout the year, as JEV transmission and infections caused by other pathogens that increase the risk of acute encephalitis syndrome may heighten during the change in seasons in some countries [13]. It also recommends that countries at risk or those that have survived JEV incursions with effective JE interventions in place carry out nationwide surveillance of laboratory-confirmed cases in all hospitals that will help healthcare systems predict the risk and growth of JEV cases and devise preventative control measures for the same in a timely manner [13]. 

## 3. AI and Current Machine-Learning Models for JEV

Artificial intelligence (AI) platforms developed through statistical algorithms and machine learning have given rise to novel ways of studying clinical risk of diseases, discovering new hallmarks of pathological states and developing robust diagnostic and prognostic markers, all of which play an important role in the design of disease management strategies and preventative measures [17,18]. Bayesian networks, commonly used in generative AI platforms, help to draw possible relationships between different variables or factors and how they influence one another in variable scenarios [19]. These networks can predict outcomes and make decisions based on the available information, and they form new knowledge in the process [20]. These capabilities enable Bayesian networks to function as an epidemiological surveillance tool for diseases, including those caused by JEV, an advantage which traditional surveillance methods that rely on limited and often specific endpoint clinical measurements appear to lack [21]. Since Bayesian networks are applied to broaden the value of clinical data obtained through traditional measurement methods of monitoring disease, the difference between the percentage accuracy and specificity of the former with that of the latter method is difficult to establish [22]. However, the high levels of accuracy of predictive AI-based modeling have been reviewed, with a 70% accuracy in predicting influenza outbreaks in Hong Kong, an 88% accuracy in predicting the risk of outbreak of three transmissible infections (Chikungunya, Malaria, and Dengue) in India, and up to a 98.44% accuracy in diagnosing parasitic infections [23]. Murty et al. (2009) input agricultural, meteorological, and animal data into a machine-learning algorithm and used the Bayesian network to calculate the density of vectors, which helped to control disease outbreaks effectively in the country [21]. The authors demonstrated that this computational approach could predict vector density (per man-hour density) of JE mosquitoes in Andhra Pradesh, where the largest number of cases in India exists, achieving an accuracy rate of up to 95%. In a different study led by Wei et al. (2023), Bayesian Information Criterion with Bernoulli prior (‘BICq’) was employed to study various risk factors (e.g., age, gender, co-existing disorders like hypertension, etc.) and analyze clinical laboratory results (e.g., blood cell counts, total protein, blood glucose, metabolite levels, etc.) measured from patients’ CSF and blood samples [18]. This clinical evaluation was utilized to categorize infected individuals into ‘good’ and ‘bad’ prognosis groups at three months post-hospital discharge [18]. Adult JE patients with increased serum neutrophil–lymphocyte ratio and percentage of neutrophils, decreased lymphocyte count, impaired glucose metabolism, and liver dysfunction are risk factors which confer a poor prognosis.

Multiple machine-learning algorithms, such as genetic, feature-selection, and OpenCV algorithms, can be combined to detect JEV infections based on the changes in the color of a cell image by comparing infected and normal healthy brain cell images and aligning these differences against patient characteristics [20]. The collective data were said to improve the detection rate of the initial infection by 96% to 99% [20]. The combined use of other algorithms is described by Ranjan et al. (2024), whose study aimed to predict the Force of Infection (FOI) which is the rate at which susceptible individuals acquire a JEV infection [24]. In this study, Ridge Regression, Lasso Regression, ElasticNet Regression, and Multilayer Perceptron were used to establish the relationship between FOI and geographical variables such as climate, rice distribution, livestock distribution, population density, specific age group density, urban/rural category, and elevation [24]. This AI model was found to have a minimum error rate and an enhanced prediction accuracy; and the output could be used to design successful interventions to limit the spread of JEV [24]. Machine-learning algorithms, including weighted correlation network analysis, can be used in conjunction with traditional laboratory techniques, such as liquid/gas chromatography–mass spectrometry, to generate protein expression profiles and molecular pathway signatures for JE [17]. Biomarkers of neuronal damage, anti-apoptosis, heat shock response, unfolded protein response, cell adhesion, and macrophage and dendritic cell activation were upregulated in the CSF of patients with JE [17]. A challenge for integrating these traditional and novel methodologies is the need to train these new algorithms and compare them to gold-standard methods for characterizing infections. This not only requires more data; it also requires establishing which existing protocols should be used to refine the machine-learning models. At present, there is no consistency across disease surveillance systems for JEV, and the parameters being investigated are often quite different.

Despite the evidence of the positive impact AI surveillance methods have made on the strategies developed to manage JEV infections and minimize the risk of outbreaks, it is imperative to recognize that machine-learning tools curate information based on data that are not only known to the researcher but also data that are publicly available without proper expert verification of facts. Hence, the information produced and conclusions drawn can be potentially biased by major factors influencing large trend changes, thus excluding any nuanced but clinically significant details that may be crucial in dissecting the various elements needed to build an effective JEV surveillance system. This limitation may be mitigated with the incorporation of both statistically validated laboratory results and AI-generated information that has been verified by JEV experts in surveillance studies to allow for holistic and meaningful interpretation of the combined data. Strategies to improve the reliability of AI tools in disease surveillance are ongoing.

## 4. Strategies to Further Develop and Improve AI Tools Through Mechanistic Modeling

Structurally, health data are siloed in different stages of healthcare provisions, such as hospitals (often for acute care), community service (often for chronic care and rehabilitation), and physicians (private and public), which often include multiple modal datasets and present barriers that limit data mobility as patients transition between settings. With the employment of the electronic health record (EHR) system, we now have a de facto health data source for models that target clinical screening, early detection, and prognosis [25]. AI algorithms can process large amounts of data faster than humans and provide more accurate results and integrate this information for a comprehensive evaluation of the health status of individuals and cross populations. For this reason, once trained and proven reliable, AI systems could improve accuracy and cost-effectiveness for JEV diagnosis, in addition to improving the targeting of preventive health campaigns to populations deemed at highest risk.

AI tools that target JE surveillance could employ computational models that vary from mathematical to mechanistic, depending on data availability and the purpose of the model. Modeling strategies could include common mathematical modeling methods used in developing AI models together with mechanistic modeling approaches tailored to JE surveillance. The combination of both machine-learning and mechanistic models has been broadly employed to improve health outcomes for various health conditions including cancer using AI-driven technologies [26]. Mathematical and machine-learning models could leverage public health data through a variety of techniques, including deep-learning models, generative models, hybrid models, and explainable AI, to predict surveillance and intervention outcomes [27]. Meanwhile, mechanistic models focus on employing health data; physiologic parameters; and principles of patient samples, such as cerebrospinal fluid (CSF) and clinical symptoms, to characterize JE detection and diagnosis and simulate interventions to improve clinical prognosis [28,29]. The integration of the two models will center on critical aspects, such as disease signature protein identification, early detection, and dynamic interactions in JE surveillance and outbreaks. This combined modeling strategy will increase the validity of the AI models produced and enable a science-informed pathway on technology application.

Mathematical models utilize deep learning to process large-scale datasets and integrate high-dimensional, multi-modal data across different stages of healthcare provisions and identify patterns across patient samples [30]. Current AI algorithms tailored to health management integrate multiple data sources into a shared embedding, mathematically modeling the similarities and dissimilarities between different data modalities, including individual personal information, clinical presentation, laboratory testing, and tabular data. The anatomy of health data construction forms the structural basis that projects subsequent tasks and can be used to generate a mathematical model to predict how changes in any of the data input, such as trends of climate change, seasonal changes, interaction with other pathogens, and population movement, may impact downstream events such as JE diagnosis, intervention, and prevention. The continuous data integration from various sources represents a clear advance in adopting AI tools in JE surveillance because this will enable the refinement and updating of AI simulation model systems that can improve the efficiency and accuracy of JE outbreak predictions in real time. Deep-learning technology has demonstrated initial success in the screening and rapid detection of various clinical conditions [31] and helps alleviate distress and prevent medical trauma pertinent to community management [32]. In cardiovascular diseases, advances in deep learning applied to electrocardiogram have enabled rapid detection of left ventricular dysfunction [33] and coronary artery disease [34]. In the context of JE, a rapid diagnosis test is urgently needed, particularly in less developed areas. Analysis of JE biomarkers often involves a large set of patient samples, followed by subsequent verification using a pipeline of protein analyses, using liquid chromatography with tandem mass spectrometry. While antibody-based assessments such as enzyme immunoassays allow for a defined analysis of JE protein signatures, it is challenging to scale up the analysis. Recently, deep machine-learning analysis of large-scale human CSF proteomics in JEV infection has shed light on the identification of JE protein signatures [17]. Strategies such as applying deep-learning models in JE data analysis could aid the development of a rapid diagnosis test in the future.

Albeit, JE possesses clear regional differences, accompanied by the imbalance of healthcare resources [3], presenting challenges in the generalization of AI tool development regarding the accuracy in AI algorithms. Therefore, region-specific adjustments such as local environmental factors, healthcare infrastructure, population density, and mobility that can influence JE risk and surveillance [35] should be incorporated into the training of AI algorithms for mathematical modeling and machine learning. Such optimization in AI algorithms could balance region-related disparities in data quality. As such, AI-driven predictions could be adopted to effectively allocate resources and mitigate bias in regions where there is a high risk with limited healthcare access. In the long run, continuous model monitoring is required to evaluate the efficiency and accuracy of AI algorithms for JE surveillance in each region. This will enable the detection of potential data drift due to changes in environmental and socioeconomical conditions [36]. Continuous retraining of AI models using updated region-specific adjustments can help optimize model accuracy across diverse settings.

## 5. Ethical Considerations and Design of Future AI Tools

AI has the potential to help epidemiologists and other public health scientists predict JEV outbreaks and identify high risk locations and populations through analyzing large amounts of data on environmental conditions, disease vector behavior, and disease transmission [37]. Given the significant burden of JEV on impacted populations, and especially children, realizing this potential has a moral dimension. Wang and Xie (2023) bring attention to the fact that JE can cause severe disability, and that the benefits of vaccination are not uniformly distributed, with underdeveloped countries often lacking the necessary resources and public health infrastructure to effectively prevent JEV infections [38]. Thus, these authors claim there is a responsibility for those countries with capacity to help thwart the virus, which will also help prevent outbreaks in what they term nontraditional epidemic areas, including Southeastern Australia [38].

When considering the use of AI in healthcare, however, there are a number of ethical issues to consider. As previously stated, bias in AI algorithms not only poses a problem for accuracy but equity as well. Distribution data on JEV infections are incomplete, particularly in high-burden countries [39]. As is the case for human decision-makers, effective AI models will need to be able to take into account the impact of underreporting and the factors influencing where this is likely to occur in order to avoid inequitable allocation of healthcare resources in a public health response. Naik et al. (2022) highlight four key ethical issues for AI use in healthcare: the need to gain informed consent for the use of health data; establishing safety and transparency standards; ensuring algorithms are fair and unbiased; and protecting data privacy [40]. These authors note that details on the design and governance of AI systems must be accessible and comprehensible to ensure clear lines of responsibility and accountability are maintained [40]. This is essential to build trust and increase the efficacy and uptake of the AI system. Ethical standards for the collection, storage, access, use, and reporting of health data, including for epidemiological surveillance, must also be established. Poor protection of patient data by public–private partnerships involved in AI development has been cited as an ethical barrier to AI research in the past, with some ethicists calling for greater regulation and oversight for big-data health research [41]. The ethical imperative to ensure fair distribution of the burdens and benefits of research also dictates that the people providing AI training data should be drawn from the population the system is set to serve in the future, and that there be no exploitation of disadvantaged citizens or their privacy to progress AI implementation in the health sector.

## 6. Discussion: Implementation of Next-Generation AI Tools and Future Studies

There is general consensus that AI has facilitated delivery of public health through spatial modeling; prediction of disease and risk; management of misinformation; and disease surveillance, spread, and diagnosis [42]. As AI continues to make strides in the healthcare sector, there is great potential to manage JE risks and surveillance and improve stakeholder engagement by embracing AI-driven technologies [43]. As aforementioned, however, the integration of AI modeling systems with health data presents clear challenges, such as trust concerns, ethical and legal considerations of algorithmic biases, and data scarcity [42]. Considering the horizon for digital tool development, strategic foresight and government support framework are required to manage critical factors and future agility for the deployment of new AI-driven tools [44].

### 6.1. Support from Government Regulation

In the USA, the application of AI tools in healthcare systems is regulated by the Food and Drug Administration (FDA) as a Medical Device [45]. Because AI is classified as a Medical Device Software (V1.0.0.0), there are frameworks for risk management and corresponding considerations based on the type of medical condition and the extent to which the software contributes to clinical management [45]. Effective governance of AI applications is a prerequisite for digital trust and data accountability while advancing the acceptance and implementation of digital tools [43]. If AI tools are to be embedded in public health management for JE, there will be a need for government regulation that establishes privacy standards for continuous and real-time management of patient samples and datasets, developing modeling systems for future and extended research over time, and promoting trust and safety in the community [46]. More broadly, an AI Act from each country could add additional measures to reduce the personal risks of AI intervention and ensure the safety of long-term implementation. This is also expected to enhance social acceptance of AI use in the healthcare setting.

### 6.2. Implementation in Public Health Systems

Implementation of an AI-driven tool within healthcare systems will require infrastructural support to store and process multi-modal large-scale data as input to AI models, as well as continuous model refinement. Moreover, health professionals and public health officers will need to be equipped with the skills regarding how to evaluate and respond to healthcare recommendations generated by AI tools. AI research has been mainly performed in non-clinical environments. The public health sector and health providers may not be ready for the implementation of AI tools in healthcare, largely due to insufficient experimental data authenticating the resource efficacy [47]. Therefore, a co-design strategy that actively engages health professionals and involves multi-disciplinary experts is recommended from the start of tool development. 

## 7. Conclusions and Future Directions

The clinical course of JE can take place in four stages, followed by long-term recovery depending on the severity of the disease [3]. The future of AI intervention in JE care will move beyond the data modeling of patient samples into expansive tools examining healthcare data and drug development in fusing multi-modal health datasets. This could enable real-time detection of JE trends from a global perspective; the discovery of JE signature proteins for rapid diagnostic tests; and the deployment of effective interventions such as new vaccines and new drug candidates to harness the disease spread. While existing vaccines for JEV demonstrate high efficacy, with studies demonstrating 85–100% seroconversion rate at the 1–3 month mark following two doses (retaining 67–92% at 1 year), the use of AI could help prioritize resources more effectively to achieve population protection even in locations with low vaccination uptake [48].

Deep-learning models and the new generative AI tools could establish a new in silico platform that better aligns JE surveillance with its diagnosis for effective intervention. Through electronic healthcare records from all health encounters, climate change data models, and government regulatory systems, AI tools may have access to millions of patient examples to develop rapid and accurate predictions on JE development and rapid detection of an epidemic. Despite these advances, however, the implementation of AI tools in JE surveillance requires evidence-based approaches with supporting data. Of particular relevance is validity testing, screening for region- or population-specific algorithmic biases, and ensuring equitable distribution of the benefits of advanced epidemiological surveillance using AI across developed and developing countries. Future AI models that incorporate physiological, environmental, and organizational data will significantly advance JE surveillance. A coordinated, multi-national effort to prioritize data and model sharing will accelerate the AI model development and the precision AI-driven JE surveillance in the digital era. 

## Data Availability

Data are contained within the article.
